# Supplementary material for: Kupffer cells control neonatal hepatic metabolism via Igf1 signaling
Source: Development. 2026 Jan 27;153(2):dev204962. doi: 10.1242/dev.204962 (PMC12891940; doi:10.1242/dev.204962)
Supplement: Supplementary information [file develop-153-204962-s1.pdf]

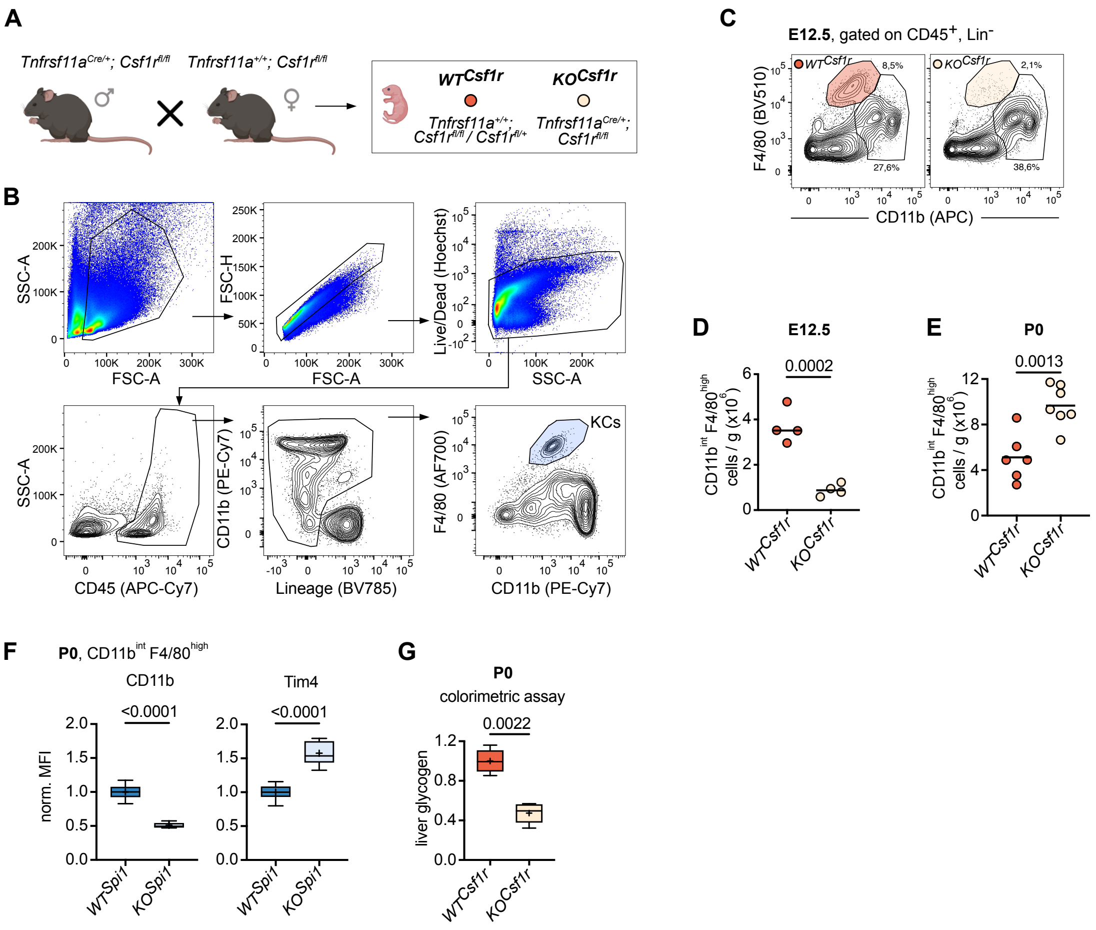

Fig. S1. Characterization of the KO^{Csf1r} mouse model. (A) Breeding scheme to produce KO^{Csf1r} and WT^{Csf1r} littermate controls. Created in BioRender by Mass, E., 2025. https://BioRender.com/jvsfc8p. This figure was sublicensed under CC-BY 4.0 terms. **(B)** Gating strategy to identify KCs in E12.5, E14.5, and P0 livers. **(C)** Representative flow cytometry plots of WT^{Csf1r} and KO^{Csf1r} fetal livers at E12.5 showing efficient depletion of KCs. **(D)** Quantification of total WT^{Csf1r} and KO^{Csf1r} KC numbers at E12.5. Circles represent individual mice. n = 4 per genotype from 2 independent litters. Unpaired Student's t-test. **(E)** Quantification of total WT^{Csf1r} and KO^{Csf1r} KC numbers at P0. Circles represent individual mice. n = 9-13 per genotype from 3 independent litters. Unpaired Student's t-test. **(F)** Normalized expression of surface receptor markers on KCs and KC-like cells from WT^{Spi1} and KO^{Spi1} mice at P0. n = 9-13 per genotype from 5 independent litters. Boxplot with 5-95 percentile. Cross indicates the mean, and the line the median. Mann-Whitney test. **(G)** Glycogen levels were measured on whole liver lysates of WT^{Csf1r} and KO^{Csf1r} at P0. n = 6 per genotype from 3 independent litters. Values were normalized per litter. Boxplot with 5-95 percentile. Cross indicates the mean, and the line the median. Mann-Whitney test.

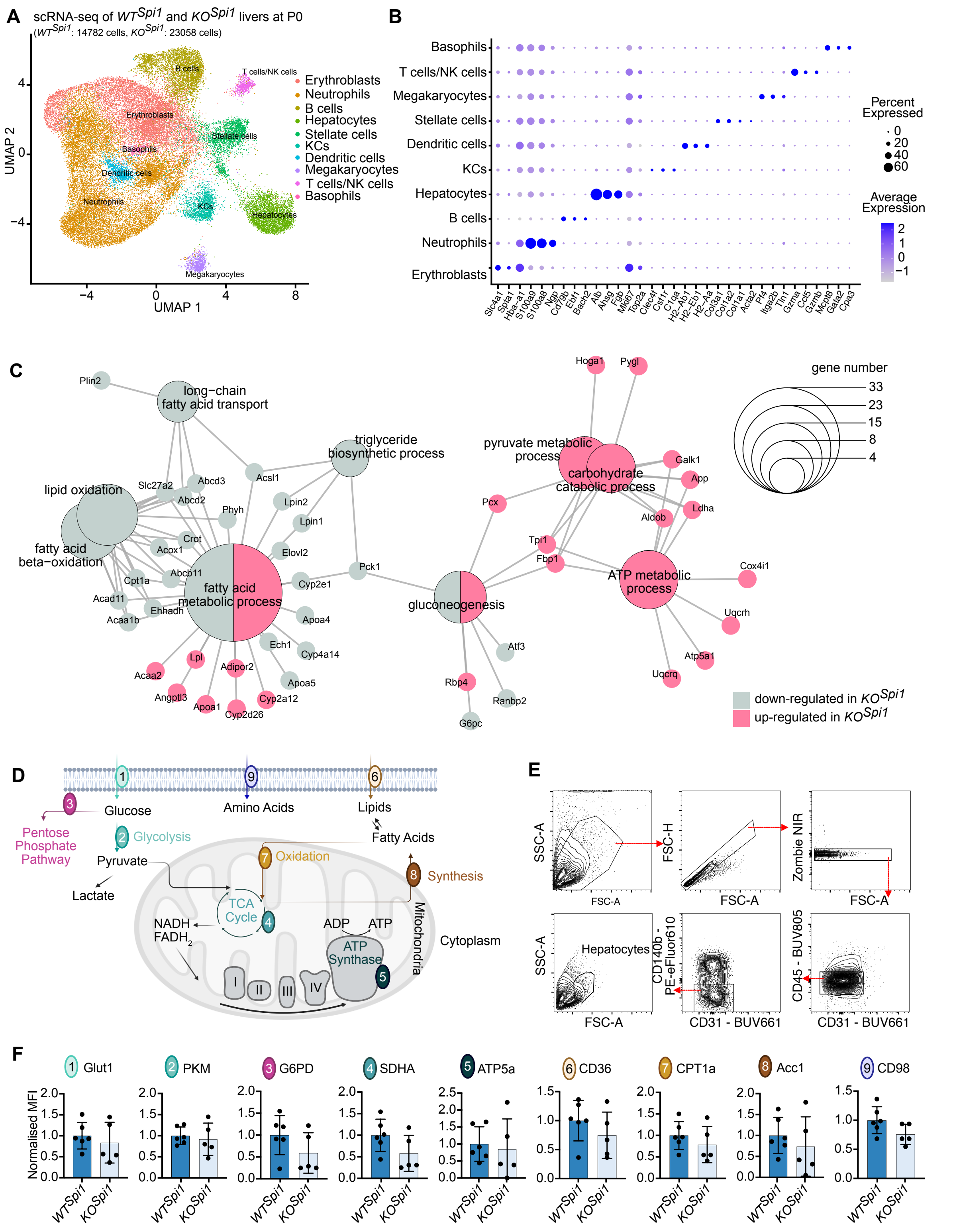

Fig. S2. scRNA-Seq analysis and metabolic profiling of the KO^{Spi1} mouse model at P0. (A) UMAP visualization of the annotated single cell clusters of WT^{Spi1} and KO^{Spi1} livers. n = 2 livers per genotype were pooled before loading cells on arrays. **(B)** Dotplot showing canonical marker genes to identify specific clusters shown in (A). **(C)** Network visualization of

gene ontology enrichment analysis of DEGs on hepatocyte cluster 2 (related to Figure 4B) showing the pathways and the involved genes. **(D)** Schematic of metabolic targets for flow cytometry-based analysis. Created in BioRender by Mass, E., 2025. https://BioRender.com/k16qpj7. This figure was sublicensed under CC-BY 4.0 terms. **(E)** Representative gating strategy of hepatocytes. **(F)** Normalized expression of metabolic targets in hepatocytes at P0 from *WT*^{Spi1} and *KO*^{Spi1}. n = 5-6 per genotype from 2 independent litters. Barplot presented as mean ± SD. Mann-Whitney test.

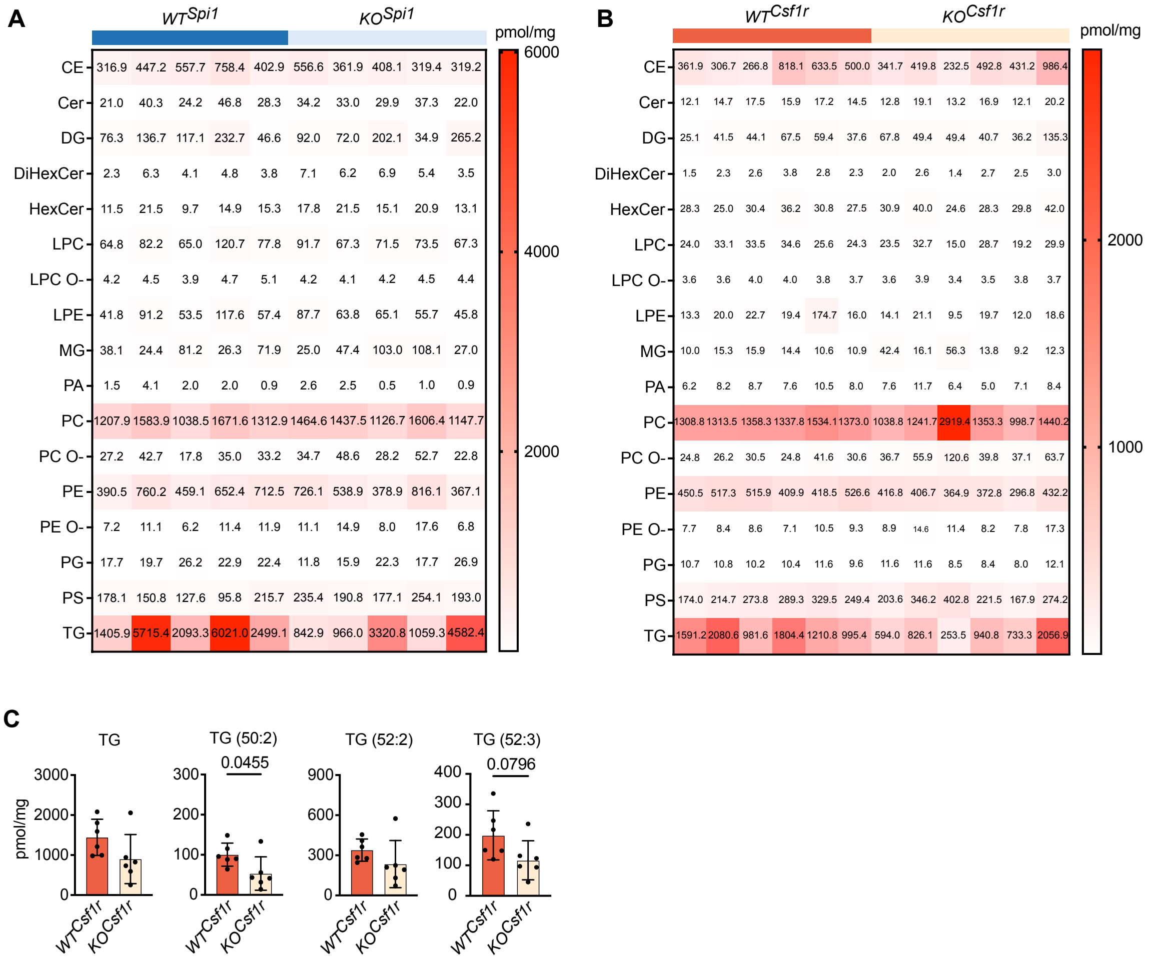

Fig. S3. Metabolic phenotyping of the KO^{Spi1} and KO^{Csf1r} mouse model. (A-B) Lipid species abundance in WT^{Spi1} and KO^{Spi1} (A) and WT^{Csf1r} and KO^{Csf1r} livers (B). n = 5-6 per genotype from more than 3 litters per mouse model. CE: Cholesteryl Ester, Cer: Ceramide, DG: Diacylglycerol, DiHexCer: Di-Hexosylceramide, HexCer: Hexosylceramide, LPC: Lysophosphatidylcholine, LPC O-: Alkyl-Lysophosphatidylcholine, LPE: Lysophosphatidylethanolamine, MG: Monoacylglycerol, PA: Phosphatidic Acid, PC: Phosphatidylcholine, PC O-: Alkyl-ether phosphatidylcholine, PE: Phosphatidylethanolamine, PE O-: Alkyl-Phosphatidylethanolamine, PG: Phosphatidylglycerol, PS: Phosphatidylserine, TG: Triacylglycerol. **(C)** The abundance of total triacylglycerol (TG) and its subspecies in WT^{Csf1r} and KO^{Csf1r} livers. n = 6 per genotype from 3 independent litters. Barplot presented as mean ± SD. Unpaired Student's t-test.

Development • Supplementary information

Table S1. Differentially expressed genes of *WT*^{Spi1} versus *KO*^{Spi1} macrophages

Available for download at
https://journals.biologists.com/dev/article-lookup/doi/10.1242/dev.204962#supplementary-data

Table S2. Gene set enrichment analysis of *WT*^{Spi1} versus *KO*^{Spi1} macrophages

Available for download at
https://journals.biologists.com/dev/article-lookup/doi/10.1242/dev.204962#supplementary-data

Table S3. GO term analysis of hepatocytes from *WT*^{Spi1} versus *KO*^{Spi1}

Available for download at
https://journals.biologists.com/dev/article-lookup/doi/10.1242/dev.204962#supplementary-data

Table S4. Phosphoproteomics of livers from *WT*^{Spi1} and *KO*^{Spi1}

Available for download at
https://journals.biologists.com/dev/article-lookup/doi/10.1242/dev.204962#supplementary-data

Table S5. Antibodies

Available for download at
https://journals.biologists.com/dev/article-lookup/doi/10.1242/dev.204962#supplementary-data
